## [Reviewer comments · Life Science Alliance]

JIP2 haploinsufficiency contributes to neurodevelopmental abnormalities in human pluripotent stem cell-derived neural progenitors and cortical neurons

Reinhard Roessler, Johanna Goldmann, Chikdu Shivalila and Rudolf Jaenisch

DOI: 10.26508/lsa.201800094

Review timeline:

Submission date:	23 May 2018
1 st Revision received:	23 May 2018
1 st Editorial Decision:	28 May 2018
2 nd Revision received:	6 June 2018
Accepted:	7 June 2018

Report:

(Note: Letters and reports are not edited. The original formatting of letters and referee reports may not be reflected in this compilation.)

Please note that the manuscript was previously reviewed at another journal and the reports were taken into account in inviting a revision for publication at Life Science Alliance prior to submission to Life Science Alliance.

Referee #1:

The authors aim to identify novel genetic contributions to Phelan-McDermid syndrome using iPSCs derived from patients containing variants of the 22q13 deletion. Although there are several genes in addition to SHANK3 included in the 22q13 deletions, this study focuses on JIP2, which has been shown in mouse knock-out models to contribute to autism-like behavior, and is known to participate in semaphorin signaling, a pathway which has also been shown to regulate neuronal maturation, likely relevant to the Phelan-McDermid disease phenotype. Their analysis of JIP2 function relies mostly on expression of semaphorin and JNK signaling proteins and target genes in NPCs and mature neurons, but demonstrates convincingly that, in addition to known signaling downstream of SHANK3, these signaling pathways are disrupted in 22q13 patient derived NPCs and neurons, and that these disruptions are due to at least one variant of the 22q13 deletion encompassing both JIP2 and SHANK3. Furthermore, they are able to demonstrate that NRP1 is a potential therapeutic target in 22q13 deletion syndrome, since JNK and semaphorin signaling can be rescued in 22q13 patient-derived neurons when the NRP1 receptor is stimulated by recombinant semaphorin. Conceptually, the identification of semaphorin signaling as a contributor to autism-like neurobiological phenotypes and establishing NRP1 as a therapeutic target would be considered a novel finding in the field (even though SEMA5A had been previously identified as a ASD risk gene; Weiss et al., 2009 Nature; this citation should be added). However, since the authors do not actually demonstrate functional contributions of JIP2/JNK or semaphorin signaling to neuronal maturation or synaptogenesis in 22q13 derived, these novel findings are still somewhat hypothetical rather than experimentally proven. Overall the paper is very well written, easy to follow and technically innovative. Additional functional analyses will strengthen the novel findings suggested by the authors.

Point by point comments:

Major concerns:

1. To establish whether or not signaling relevant to known functions of JIP2 are important in 22q13 deletion NPCs and neurons, they look at expression of proteins in the semaphorin signaling pathway, and find that this pathway is indeed disrupted in the iPSC-derived neurons, and to a lesser extent in the NPCs. Pharmacological stimulation of this pathway with the NRP1 ligand semaphorin is able to reverse the protein expression changes in the semaphorin pathway observed in the iPSC derived neurons. Identifying the semaphorin pathway as being perturbed in 22q13 deletion syndrome is a novel finding and provides a potential therapeutic target, in this case demonstrated by stimulation of semaphorin signaling. However, it is surprising that the authors do not perform any functional analyses to really show what contribution the changes in JNK signaling have to the final phenotype, which in this manuscript consists of neuronal maturation and synaptogenesis defects. This could be done by analyzing neuronal maturation and synaptogenesis in 22q13 iPSC derived neurons after rescue of semaphorin signaling. Or, to better delineate the contribution of JIP2 specifically, the authors could choose to rescue JIP2 expression in the iPSC derived neurons, and then determine the effects of this rescue on neuronal maturation and/or synaptogenesis. The way the paper is written, the authors seem to assume that changes in JNK signaling are directly causing the neuronal maturation effects, but do not actually demonstrate the connection between JNK signaling and neuronal maturation in the mutant cells. In the discussion the authors claim that they have added mechanistic insights into the 22q13 deletion syndrome beyond loss of SHANK3, however without demonstrating a functional rescue or single gene knock-out strategy these insights remain speculative. Although semaphorin

signaling has been implicated in neuronal maturation, in this case, where multiple genes are deleted, one of which (SHANK3) directly impacts the functional phenotype analyzed (EP), a functional rescue needs to be combined with stimulation of semaphorin signaling, if that is the main point of the paper, or with rescue of JIP2, if the main point of the paper is the contribution of JIP2 specifically.

Minor concerns:

- Figure 1C-E. Based on the Western Blots shown in 1C from patient 1, it looks like there is still quite a lot of Pax6 coincident with decreased NeuN and possibly DCX, which could be evidence for delayed differentiation in addition to delayed maturation. How much of this could be due to using ESCs instead of iPSCs as the control population? The propensity for neuronal differentiation differs between different ESC lines and between iPSC and ESC lines.
- Figure 1C data is labeled as patient 1, but in Figure 4D it looks like the effects on JNK signaling is more pronounced in patient 2 cells. Based on Table 1, the patient 2 deletion is much larger but appears to only include JIP2, ARSA and SHANK3 according to Figure 3A, whereas Figure 2H shows several genes upstream of JIP2 and downstream of SHANK3 included in the deletion. It is not really clear which of the patient lines is being used for each of these experiments, and actually patient 2 in comparison to patient 1 might be helpful in terms of arguing for JIP2-specific effects. Please clarify.
- In the discussion the authors claim that loss of JIP2 coincides with reduced JNK expression in NPCs, however in Figure 4B JNK expression is not effected in the iPSC-derived NPCs.
- The methods section states that the controls used as a comparison to the patient induced PSCs were human ESC cell line WIBR, but in the text for (line 4 of the first results section) they state that control PSCs were used. Please clarify whether or not wild-type iPSCs were used as controls for the patient iPSCs rather than wild-type hESCs.
- There are a few typos in the text ("know" should be "known" in line 5 paragraph 2 of intro, a mistake that is replicated in several other places), there are two // instead of one / in Figure 3G JNK 1/3 label under the graph
- In the methods is stated that the NPCs were analyzed 14-16 days after Fgf2 withdrawal, this is surprising since Fgf2 withdrawal usually leads to neuronal differentiation within days. Is this correct? Or did the authors mean that early stage (immature) neurons were analyzed at this time?

Referee #2:

Comments on "JIP2 haploinsufficiency contributes to neurodevelopmental abnormalities in human pluripotent stem cell-derived neural progenitors and cortical neurons"

In this work, Roessler et al suggest that 22q13 Deletion Syndrome, which is believed to be predominantly caused by heterozygous deletion of the SHANK3 gene, might additionally be linked to the loss of JIP2. They reprogram fibroblasts from 22qDS patients and use genome editing techniques, and show an impaired neurodevelopment and neuronal maturation in patient-derived neurons. The authors also show impaired phenotypes in isogenic ESC-derived neurons which they created. Finally, they showed that pharmacological stimulation ultimately elevated JNK protein levels which were reduced in patient-derived neurons and thought to be one of the causes of the impaired generation of mature neurons.

While this paper potentially provides new mechanistic and genetic insights on the 22q13 Deletion Syndrome, there are several major concerns with some of the key findings presented in this manuscript.

The main claim of this paper, as the title suggests, is that JIP2 contributes to the neurodevelopmental abnormalities in patient-derived neurons and in genome edited isogenic lines. However, the authors do not show its direct involvement in the process. JIP2 contribution should be tested on a deletion that includes SHANK3, but excludes other genes. Furthermore, instead of a pharmacological stimulation which was intended to alleviate the phenotype, allegedly caused by JIP2 deletions and misregulation of JNK proteins, the authors should have designed a rescue experiment, where they show a correction of the phenotype via JIP2 expression.

Another major concern is that in the first two figures the authors compare patient-derived to ESC-derived NPC and neurons, which is not the appropriate control. For such non-isogenic controls more cell lines are required. Isogenic lines would provide a more satisfactory and definite proof for the claims. Also, the isogenic lines the authors present later, lack description and information regarding their background, their indels, and the second allele. They also present a case where the entire locus is deleted rather than JIP2 alone (or else rescue of JIP2 in the background of the deletion).

Finally, the PAX6-GFP reporter the authors designed (Figure 2), is also a bit flawed. While PAX6 is a known marker for NPC, Figure 1 indicates that it is expressed to a greater extent in the immature neurons rather than in the NPC, making the comparison problematic.

Additional concerns:

Figure 1

- A and B should be compared to patient iPSC
- Nestin is very abundant in A but not in C
- In page 4 the authors state "...a severe reduction of neuronal markers such as DCX" (D) but this reduction is not significant let alone "severe"
- Elaboration is needed in the text for the term "neuron content" in E

Figure 2

- When compared to supplementary figure 2. F, H seems not significant

Figure 4

- In the text (page 8), "Strikingly, we observed an increase in NRP1 transcript in 22qDS NPCs (Fig.4B)". Statistical

analysis is missing.
- On page 9, Fig.4E instead of Fig.4D

Referee #3:

This is a rather confused paper that collects various datasets, but lacks coherence. Overall, the paper contains some interesting individual experiments, but the paper does not substantiate the claims made in the abstract. In places, the experiments are notably underpowered.

Specific comments:

The authors carry out experiments on 22q13 iPSCs, generating neurons and recording neuronal activity on MEAs. The authors conclude the the patient iPSC-derived neurons have delayed maturation, based on MEAs. However, electrical maturation is highly variable among stem cell cultures, so this is not a robust readout. Furthermore, the reasons for lack of readout from individual electrodes include glial overgrowth, altering electrode contact. At the very least, the authors would need single neuron recordings, or an independent method to record neuronal activity, to confirm this phenotype.

The second figure reports profiling of Pax6+ progenitor cells, using a reporter. This construct would appear to disrupt one Pax6 allele, making the cells Pax6 heterozygotes. Pax6 haploinsufficiency is known to alter forebrain development, including neurogenesis, which makes these experiments challenging to interpret. How did the authors control for this? Notwithstanding this, the analysis of neurogenesis quite superficial and seems to rest entirely on transcriptomics.

The third figure details generation of CRISPR-driven deletions in the region of 22q13. Other than generating lines, it is not clear what the conclusion is here - the authors state that they observe disease-specific phenotypes, but other than a loss of SHANK3, it was not clear what they were referring to here.

Finally, there is a section on semaphorin signalling. Again, it is not clear why this experiment was performed. There is no real characterisation of the biological consequences of JIP2 deficiency, for example. The experiments on manipulating sema signaling led to some changes in NRP1 levels, but, again, the biological context here is confused.

1st Revision – authors' response

23 May 2018

Referee #1 (Report for Author)

The authors aim to identify novel genetic contributions to Phelan-McDermid syndrome using iPSCs derived from patients containing variants of the 22q13 deletion. Although there are several genes in addition to SHANK3 included in the 22q13 deletions, this study focuses on JIP2, which has been shown in mouse knock-out models to contribute to autism-like behavior, and is known to participate in semaphorin signaling, a pathway which has also been shown to regulate neuronal maturation, likely relevant to the Phelan-McDermid disease phenotype. Their analysis of JIP2 function relies mostly on expression of semaphorin and JNK signaling proteins and target genes in NPCs and mature neurons, but demonstrates convincingly that, in addition to known signaling downstream of SHANK3, these signaling pathways are disrupted in 22q13 patient derived NPCs and neurons, and that these disruptions are due to at least one variant of the 22q13 deletion encompassing both JIP2 and SHANK3. Furthermore, they are able to demonstrate that NRP1 is a potential therapeutic target in 22q13 deletion syndrome, since JNK and semaphorin signaling can be rescued in 22q13 patient-derived neurons when the NRP1 receptor is stimulated by recombinant semaphorin. Conceptually, the identification of semaphorin signaling as a contributor to autism-like neurobiological phenotypes and establishing NRP1 as a therapeutic target would be considered a novel finding in the field (even though SEMA5A had been previously identified as a ASD risk gene; Weiss et al., 2009 Nature; this citation should be added)...Ref has been added. However, since the authors do not actually demonstrate functional contributions of JIP2/JNK or semaphorin signaling to neuronal maturation or synaptogenesis in 22q13 derived, these novel findings are still somewhat hypothetical rather than experimentally proven. Overall the paper is very well written, easy to follow and technically innovative. Additional functional analyses will strengthen the novel findings suggested by the authors.

Major concerns:

1. To establish whether or not signaling relevant to known functions of JIP2 are important in 22q13 deletion NPCs and neurons, they look at expression of proteins in the semaphorin signaling pathway, and find that this pathway is indeed disrupted in the iPSC-derived neurons, and to a lesser extent in the NPCs. Pharmacological stimulation of this pathway with the NRP1 ligand semaphorin is able to reverse the protein expression changes in the semaphorin pathway observed in the iPSC derived neurons. Identifying the semaphorin pathway as being perturbed in q22.13 deletion syndrome is a novel finding and provides a potential therapeutic target, in this case demonstrated by stimulation of semaphorin signaling. However, it is surprising that the authors do not perform any functional analyses to really show what contribution the changes in JNK signaling have to the final phenotype, which in this manuscript consists of neuronal maturation and synaptogenesis defects. This could be done by analyzing neuronal maturation and synaptogenesis in 22q13 iPSC derived neurons after rescue of semaphorin signaling.

Response: The experiments suggested by the reviewer are very valuable and would certainly improve the quality of our manuscript. Analyzing neuronal maturation by e.g. multi-electrode array analysis or classical e-phys experiments would serve as straight-forward evaluation of the effects of semaphoring signaling rescue, however, performing all necessary experiments with all generated iPSC lines would easily exceed a 3 month time frame. Nonetheless, we have addressed these concerns and the need for future studies in the discussion.

Or, to better delineate the contribution of JIP2 specifically, the authors could choose to rescue JIP2 expression in the iPSC derived neurons, and then determine the effects of this rescue on neuronal maturation and/or synaptogenesis. The way the paper is written, the authors seem to assume that changes in JNK signaling are directly causing the neuronal maturation effects, but do not actually demonstrate the connection between JNK signaling and neuronal maturation in the mutant cells.

Response: Genetic rescue experiments to re-establish JIP2 expression (potentially in an inducible fashion) would allow us to show causality directly. Including such experiments would certainly improve the current manuscript. However, these long-term gene editing experiments are somewhat out of the scope of this initial study describing a potential role for JIP2 in 22qDS. We addressed some of these concerns in the discussion.

In the discussion the authors claim that they have added mechanistic insights into the 22q13 deletion syndrome beyond loss of SHANK3, however without demonstrating a functional rescue or single gene knock-out strategy these insights remain speculative.

Response: We have changed the manuscript accordingly and have toned down our conclusions.

Although semaphorin signaling has been implicated in neuronal maturation, in this case, where multiple genes are deleted, one of which (SHANK3) directly impacts the functional phenotype analyzed (EP), a functional rescue needs to be combined with stimulation of semaphorin signaling, if that is the main point of the paper, or with rescue of JIP2, if the main point of the paper is the contribution of JIP2 specifically.

Response: We appreciate the value of the proposed functional rescue. However, while functional rescue experiments would be conceptually straight-forward, we are currently not able to add the required experiments. We acknowledge the need for these experiments in future studies in the manuscript.

Minor concerns:

- Figure 1C-E. Based on the Western Blots shown in 1C from patient 1, it looks like there is still quite a lot of Pax6 coincident with decreased NeuN and possibly DCX, which could be evidence for delayed differentiation in addition to delayed maturation. How much of this could be due to using ESCs instead of iPSCs as the control population? The propensity for neuronal differentiation differs between different ESC lines and between iPSC and ESC lines.

Response: A direct comparison of ESCs and iPSCs is certainly not optimal, however comparing genetically unmatched iPSCs (e.g. multiple CTRL vs. patient lines) and their progeny is likely to result in equal discrepancies from line to line as has been proposed in e.g. Choi et al., 2015. Ideally, all experiments should be repeated with isogenic lines including SHANK3, JIP2 and SHANK3/JIP-deletion lines. The establishment of these lines and the necessary follow-up experiments would however by far exceed acceptable time frames.

- Figure 1C data is labeled as patient 1, but in Figure 4D it looks like the effects on JNK signaling is more pronounced in patient 2 cells. Based on Table 1, the patient 2 deletion is much larger but appears to only include JIP2, ARSA and SHANK3 according to Figure 3A, whereas Figure 2H shows several genes upstream of JIP2 and downstream of SHANK3 included in the deletion. It is not really clear which of the patient lines is being used for each of these experiments, and actually patient 2 in comparison to patient 1 might be helpful in terms of arguing for JIP2-specific effects. Please clarify.

Response: Table 1 shows that the patient 1 (22q13_1) carries a 481.187bp deletion vs. patient 2 (22q13_2) how carries a 93.894bp deletion. The deletion in patient 2 affects only JIP2, ARSA and SHANK3 as indicated in the manuscript. We clarified patient ID's in the table.

- The methods section states that the controls used as a comparison to the patient induced PSCs were human ESC cell line WIBR, but in the text for (line 4 of the first results section) they state that control PSCs were used. Please clarify whether or not wild-type iPSCs were used as controls for the patient iPSCs rather than wild-type hESCs.

Response: With "PSCs" we refer to pluripotent stem cells including both ESCs and iPSCs. Our control cells are "wild-type" ESCs. All generated lines are listed and described in table 1.

- There are a few typos in the text ("know" should be "known" in line 5 paragraph 2 of intro, a mistake that is replicated in several other places), there are two // instead of one / in Figure 3G JNK 1/3 label under the graph

Response: We have changed the manuscript accordingly.

• In the methods is stated that the NPCs were analyzed 14-16 days after Fgf2 withdrawal, this is surprising since Fgf2 withdrawal usually leads to neuronal differentiation within days. Is this correct? Or did the authors mean that early stage (immature) neurons were analyzed at this time?

Response: This refers to 14-16 days after neural induction.

Referee #2 (Report for Author)

Comments on "JIP2 haploinsufficiency contributes to neurodevelopmental abnormalities in human pluripotent stem cell-derived neural progenitors and cortical neurons"

In this work, Roessler et al suggest that 22q13 Deletion Syndrome, which is believed to be predominantly caused by heterozygous deletion of the SHANK3 gene, might additionally be linked to the loss of JIP2. They reprogram fibroblasts from 22qDS patients and use genome editing techniques, and show an impaired neurodevelopment and neuronal maturation in patient-derived neurons. The authors also show impaired phenotypes in isogenic ESC-derived neurons which they created. Finally, they showed that pharmacological stimulation ultimately elevated JNK protein levels which were reduced in patient-derived neurons and thought to be one of the causes of the impaired generation of mature neurons.

While this paper potentially provides new mechanistic and genetic insights on the 22q13 Deletion Syndrome, there are several major concerns with some of the key findings presented in this manuscript.

The main claim of this paper, as the title suggests, is that JIP2 contributes to the neurodevelopmental abnormalities in patient-derived neurons and in genome edited isogenic lines. However, the authors do not show its direct involvement in the process. JIP2 contribution should be tested on a deletion that includes SHANK3, but excludes other genes.

Response: We appreciate the suggestion to analyze consequences of JIP2 deletion only and or combined with SHANK3. Ideally, all experiments should be repeated with isogenic lines including SHANK3, JIP2 and SHANK3/JIP-deletion lines. The establishment of these lines and the necessary follow-up experiments would however by far exceed acceptable time frames. We have added a sentence in the manuscript to acknowledge the value of additional PSC pairs.

Furthermore, instead of a pharmacological stimulation which was intended to alleviate the phenotype, allegedly caused by JIP2 deletions and misregulation of JNK proteins, the authors should have designed a rescue experiment, where they show a correction of the phenotype via JIP2 expression.

Response: The reviewer points out reasonable genetic rescue experiments, which would certainly be informative. Such experiments to re-establish JIP2 expression (potentially in an inducible fashion) would allow us show causality directly. Unfortunately, we are not able to perform such cumbersome experiments in addition to the provided data.

Another major concern is that in the first two figures the authors compare patient-derived to ESC-derived NPC and neurons, which is not the appropriate control. For such non-isogenic controls more cell lines are required. Isogenic lines would provide a more satisfactory and definite proof for the claims. Also, the isogenic lines the authors present later, lack description and information regarding their background, their indels, and the second allele. They also present a case where the entire locus is deleted rather than JIP2 alone (or else rescue of JIP2 in the background of the deletion).

Response: A direct comparison of ESCs and iPSCs is certainly not optimal, however comparing genetically unmatched iPSCs (e.g. multiple CTRL vs. patient lines) and their progeny is likely to result in equal discrepancies from line to line as has been proposed in e.g. Choi et al., 2015. As indicated above, ideally, all experiments should be repeated with isogenic lines including SHANK3, JIP2 and SHANK3/JIP-deletion lines. We have adapted the manuscript to highlight the value and necessity of these lines in future studies.

Finally, the PAX6-GFP reporter the authors designed (Figure 2), is also a bit flawed. While PAX6 is a known marker for NPC, Figure 1 indicates that it is expressed to a greater extent in the immature neurons rather than in the NPC, making the comparison problematic.

Response: As indicated above, line to line discrepancies are inevitable, however we attempted to synchronize our differentiation procedure as far as possible. While PAX6 expression persist in immature neurons our RNAseq data analysis in our purified PAX6-GFP positive cell however reveals a clear NPC expression pattern (see figure 2G).

Additional concerns:

Figure 1

- A and B should be compared to patient iPSC
- Nestin is very abundant in A but not in C
- In page 4 the authors state "...a severe reduction of neuronal markers such as DCX" (D) but this reduction is not significant let alone "severe"
- Elaboration is needed in the text for the term "neuron content" in E

Figure 2

- When compared to supplementary figure 2. F, H seems not significant

Figure 4

- In the text (page 8), "Strikingly, we observed an increase in NRP1 transcript in 22qDS NPCs (Fig.4B)". Statistical analysis is missing.
- On page 9, Fig.4E instead of Fig.4D

Response: We have changed the manuscript accordingly.

Referee #3 (Report for Author)

This is a rather confused paper that collects various datasets, but lacks coherence. Overall, the paper contains some interesting individual experiments, but the paper does not substantiate the claims made in the abstract. In places, the experiments are notably underpowered.

Specific comments:

The authors carry out experiments on 22q13 iPSCs, generating neurons and recording neuronal activity on MEAs. The authors conclude the the patient iPSC-derived neurons have delayed maturation, based on MEAs. However, electrical maturation is highly variable among stem cell cultures, so this is not a robust readout. Furthermore, the reasons for lack of readout from individual electrodes include glial overgrowth, altering electrode contact. At the very least, the authors would need single neuron recordings, or an independent method to record neuronal activity, to confirm this phenotype.

Response: We appreciate the suggested experiments. Including additional cell lines (including isogenic pairs) would clearly increase the robustness of our current data set. However, generation of all needed lines and execution of all necessary experiment is currently impossible. We realize that additional, more comprehensive experiments such as single neuron recordings would improve the current form of our manuscript.

The second figure reports profiling of Pax6+ progenitor cells, using a reporter. This construct would appear to disrupt one Pax6 allele, making the cells Pax6 heterozygotes. Pax6 haploinsufficiency is known to alter forebrain development, including neurogenesis, which makes these experiments challenging to interpret. How did the authors control for this? Notwithstanding this, the analysis of neurogenesis quite superficial and seems to rest entirely on transcriptomics.

The third figure details generation of CRISPR-driven deletions in the region of 22q13. Other than generating lines, it is not clear what the conclusion is here - the authors state that they observe disease-specific phenotypes, but other than a loss of SHANK3, it was not clear what they were referring to here.

Response: As in the patient lines we observed reduced expression of JNK and DCX, which we argue is the consequence of an impaired neuronal maturation in patient neurons.

Finally, there is a section on semaphorin signalling. Again, it is not clear why this experiment was performed. There is no real characterisation of the biological consequences of JIP2 deficiency, for example. The experiments on manipulating sema signaling led to some changes in NRP1 levels, but, again, the biological context here is confused.

Thank you for submitting your revised manuscript entitled "JIP2 loss contributes to abnormalities in human PSC-derived neural progenitors and cortical neurons". Your manuscript was previously reviewed at a different journal, and the reviewer reports were confidentially transferred to us. You submitted a point-by-point response and a revised manuscript to Life Science Alliance. We have now assessed your revised manuscript and the response to the reviewers you provided.

The reviewers of the previous round of review were concerned that your conclusions were not sufficiently supported by the data provided. However, they also noted that your findings would be of value to the field and open avenues for further research. Given this and the fact that you significantly toned-down your previous conclusions in the revised version of your manuscript, we would be happy to publish it in Life Science Alliance.

Thank you for submitting your Research Article entitled "JIP2 loss contributes to abnormalities in human PSC-derived neural progenitors and cortical neurons". It is a pleasure to let you know that your manuscript is now accepted for publication in Life Science Alliance. Congratulations on this interesting work.